# Supplementary material for: Safety and Activity of the Combination of Ceritinib and Dasatinib in Osteosarcoma
Source: Cancers (Basel). 2020 Mar 26;12(4):793. doi: 10.3390/cancers12040793 (PMC7225940; doi:10.3390/cancers12040793)
Supplement: Supplementary file 1 [file cancers-12-00793-s001.zip › cancers-744773 supplementary final/cancers-744773 supplementary final.pdf]

Supplementary Materials:

a

	Analysis on paraffin embedded primary OS cells	Staining	Immunocytochemistry	No 386	No 403
Cytology	Cell density	HE (Hematoxylin and eosin)		moderate	moderate
	Cell atypia	HE		present	present
	Osteoid formation	Goldner		present	Not present
Lineage differentiation	Mesenchymal Cell		Vimentin	High positive	High positive
	Endothelium		CD31	negative	negative
	Smooth muscle		Actin	negative	negative
	Striated muscles		Desmin	negative	negative
	Macrophages		CD68	negative	negative
	Pan leukocytes		CD45	negative	negative
	Epithelium		Cytokeratin (MNF)	negative	negative
			Cytokeratin (AE 1/3)	negative	negative
			Cytokeratin (OSCAR)	negative	negative

b c

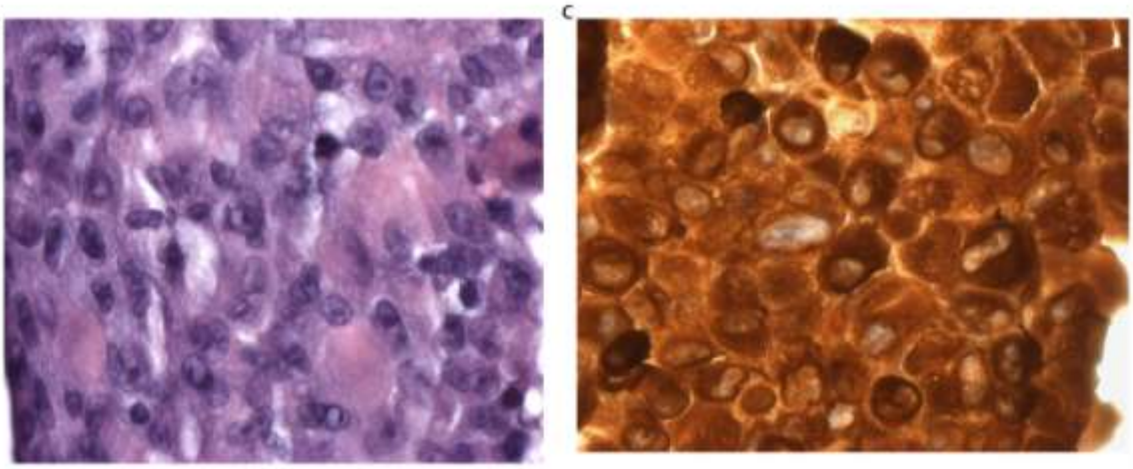

Figure S1. Morphological and immunocytochemical characterization of primary OS cells. (**a**) Strategy used to characterize the primary OS cells. Results for samples no 386 and no 403 are shown. (**b**) HE Staining (×630) and (**c**) Vimentin staining (×630) of sample no 386.

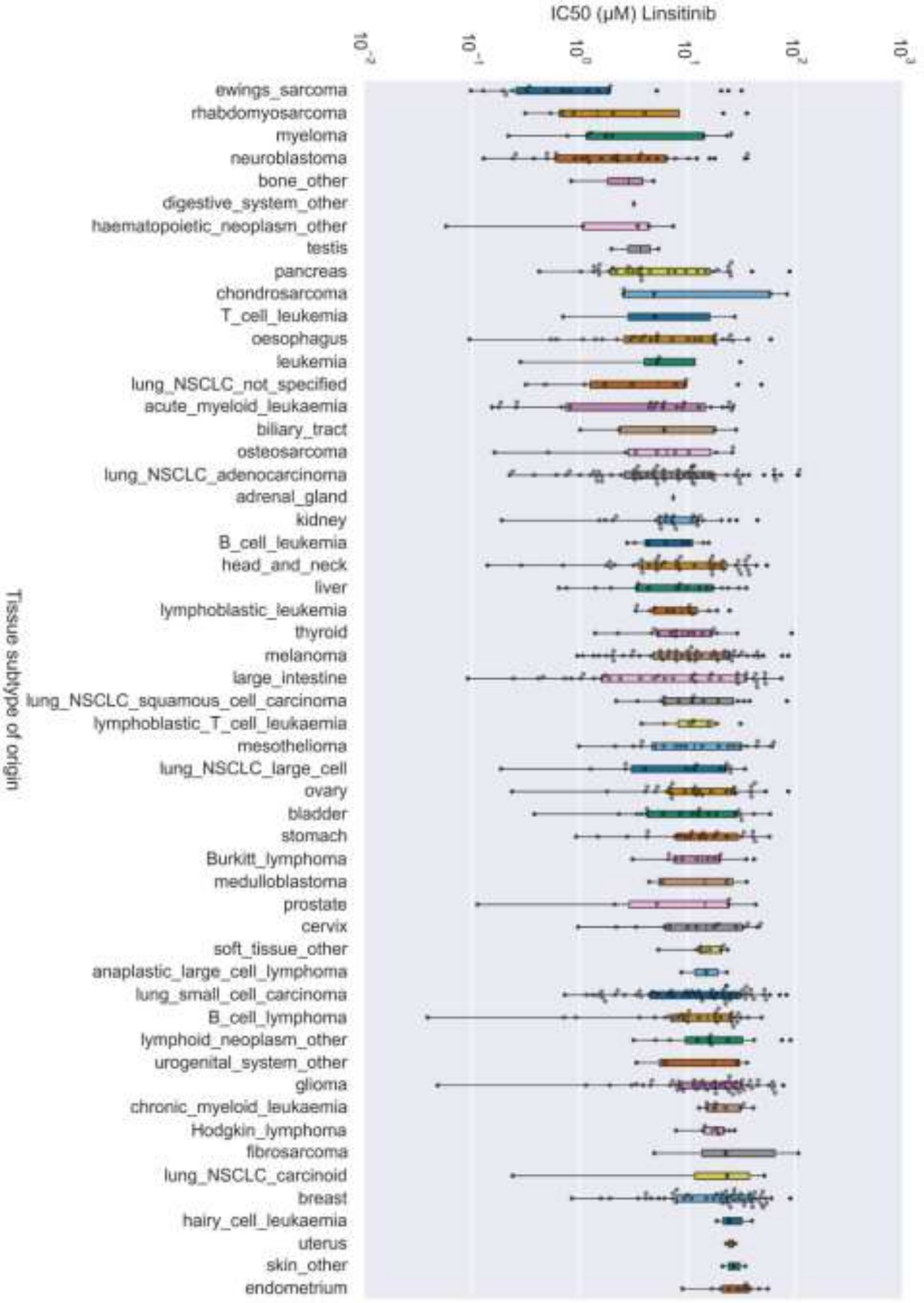

Figure S2. IC_{50} of Linsitinib in Cell lines. Boxplot (colored) and the corresponding swarmplot (Grey dots) in the background. Black dots indicate outliers. The axis on top shows the molarity needed to reach a half maximal inhibitory concentration (IC_{50}). The Tissue sub-types are in order of their median, from smallest to greatest. A small IC50 indicating a strong impact of the tested compound (Linsitinib) on the cell growth of all tested cell lines. The plot was produced with a Python package called Seaborn. The data was taken from the GDSC (Genomics of Drug Sensitivity in Cancer) Screening Set 1 of the Cancer genome Project at the Wellcome Sanger Institute and the Center for Molecular Therapeutics at the Massachusetts General Hospital Cancer Center. Information on the Screenings workflow and the established cell lines can be found at https://www.cancerrxgene.org/.

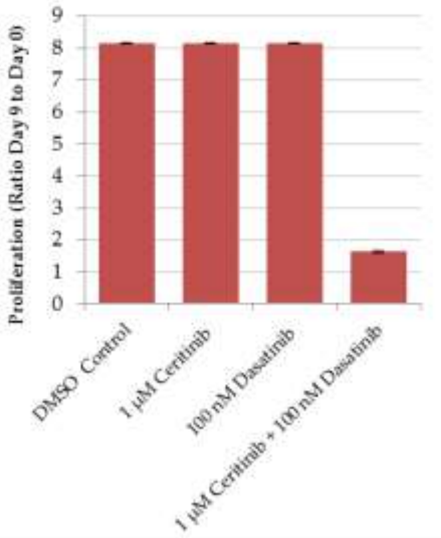

Figure S3. Effect of ceritinib and dasatinib on the proliferation of the primary tumor cells no 403. The primary tumor cells of sample 403 were incubated with the indicated inhibitors. After nine days, the cell proliferation was measured and the ratio between the absorbance at day 9 and the absorbance at day 0 was calculated. The proliferation experiments were carried out in biological duplicates. Data are represented as the mean ± Standard deviation (SD).

Table S1. Analysis performed to characterise the OS primary cells.

	Analysis on paraffin embedded primary OS cells	Staining	Immunocytochemistry
**Cytology**	Cell density	HE (Hematoxylin and eosin)	
	Cell atypia	HE	
	Osteoid formation	Goldner	
**Lineage differentiation**	Mesenchymal Cell		Vimentin
	Endothelium		CD31
	Smooth muscle		Actin
	Striated muscles		Desmin
	Macrophages		CD68
	Pan leukocytes		CD45
	Epithelium		Cytokeratin (MNF)<br>Cytokeratin (AE 1/3)<br>Cytokeratin (OSCAR)

Table S2. Primer used for *ALK* sequencing

Primer	Sequence (5´-3´)
ALK_Ex1_1_vor	TCAGAGCCTCTTCCCATCTC
ALK_Ex1_1_rev	TTCAGCACCCTGGACAGC
ALK_Ex1_2_vor	GCTGCTCAGGTTGCTGG
ALK_Ex1_2_rev	GGTGGAAGTGAAGATTATTTAATGG
ALK_Ex2_vor	GCTAAAAGACACACCCCAAGC
ALK_Ex2_rev	TCCCAAACTGAAACTCACAGAG
ALK_Ex3_vor	GCCAACCTCCTAGTGTGGAG
ALK_Ex3_rev	TATTCCAGCCTGGCCCTG
ALK_Ex4_vor	CCACAGAGCTACTGCTGGTC
ALK_Ex4_rev	ACCAAAAGCCAAATCACCTG
ALK_Ex5_vor	GTGCCCATGAAGAATGTGAG
ALK_Ex5_rev	TAAGTGTGCACATTCCCAGC
ALK_Ex6_vor	GGACCAACCCATCAGGC
ALK_Ex6_rev	GGGTTATGAGCATGGGCTG
ALK_Ex7_vor	TAATTCGACTTGGCATTGGC
ALK_Ex7_rev	TCCCATCTGTCTATGTGGGC
ALK_Ex8_vor	TGCACAGTCCATTAACCCTG
ALK_Ex8_rev	AAGTGACAAGAGGTGGGAGG
ALK_Ex9_vor	CAGTGGAGAATTCCAGAAAGG
ALK_Ex9_rev	TGTGTGTCTTGGGTAAAAGGC
ALK_Ex10-11_vor	TGCTGGATGCTGAATCCTAAG
ALK_Ex10-11_rev	GCACCAATCTTTCTTCTGCC
ALK_Ex12_vor	AGATATCTCAGAGGCCACCC
ALK_Ex12_rev	CAGGCTTCTTCGGAAGGG
ALK_Ex13_vor	GGAAGCTGCTGGGTTGG
ALK_Ex13_rev	CTCCAAGAGGCCTTCCC
ALK_Ex14_vor	TTCTGTCTGCTGCAAAGTGG
ALK_Ex14_rev	CCAGGGCTGTCATGAGG
ALK_Ex15_vor	TTTACCAGAGCTTAGGGGCTC
ALK_Ex15_rev	AGCTGAAGGCCTGGGAG
ALK_Ex16_vor	CCTGCATCCTGCTTCTCTG
ALK_Ex16_rev	TGAGGAGCCTAGGACTAAGCAG
ALK_Ex17_vor	TTAAGATTTTGGCACCCACC
ALK_Ex17_rev	CTTGGTGGGAGGACTGACC
ALK_Ex18_vor	ACTGAGGCTGGGAGTGGG
ALK_Ex18_rev	AAACCATTTGTGGTCATGGG
ALK_Ex19_vor	TTGGGACCAACTCAAAGGAG

ALK_Ex19_rev	CACTATTCAGTCCTGCCTTCC
ALK_Ex20_vor	TTCCTGTAGGAAGTGGCCTG
ALK_Ex20_rev	AACATTCAGCCCCTACACTG
ALK_Ex21-22_vor	GCCTCATTATTGTGGCCTG
ALK_Ex21-22_rev	GGAGATATCGATCTGTTAGAAACC
ALK_Ex23_vor	TTCCTCCCAGTTTAAGATTTGC
ALK_Ex23_rev	CTTCCATCCTTGCTCCTGTC
ALK_Ex24_vor	AGCATTTCAGATTTCCCTCC
ALK_Ex24_rev	CTTGAGATCTGCGGGGAAG
ALK_Ex25_vor	GTGATGGCCGTTGTACACTC
ALK_Ex25_rev	ATTCTTGAGGGGCTGAGGTG
ALK_Ex26_vor	AGCAGGGCAGATGCTTAATG
ALK_Ex26_rev	CCAGGAGCACCACCTTATG
ALK_Ex27_vor	AATGTGGGTGGGTGTGTC
ALK_Ex27_rev	AAGAAGCATATGTGGCTCTGG
ALK_Ex28_vor	ACGTATTCGTTGCAACCCTG
ALK_Ex28_rev	TTGTACTCTGACTGGCTTGACC
ALK_Ex29_1_vor	TTTGTATGCCCCAGTGCC
ALK_Ex29_1_rev	CAGTACAGCTTCCCTCCAGC
ALK_Ex29_2_vor	TTCTCTCAGTCCAACCCTCC
ALK_Ex29_2_rev	CTCTGGTTTGTGAAGGAGCC
ALK1_Ex29_3_vor	CCTGGAGCTGGTCATTACG
ALK1_Ex29_3_rev	CTCAAATGGCTCATGTCCAC
