# Supplementary figures and images for: Safety and Activity of the Combination of Ceritinib and Dasatinib in Osteosarcoma
Source: Cancers (Basel). 2020 Mar 26;12(4):793. doi: 10.3390/cancers12040793 (PMC7225940; doi:10.3390/cancers12040793)

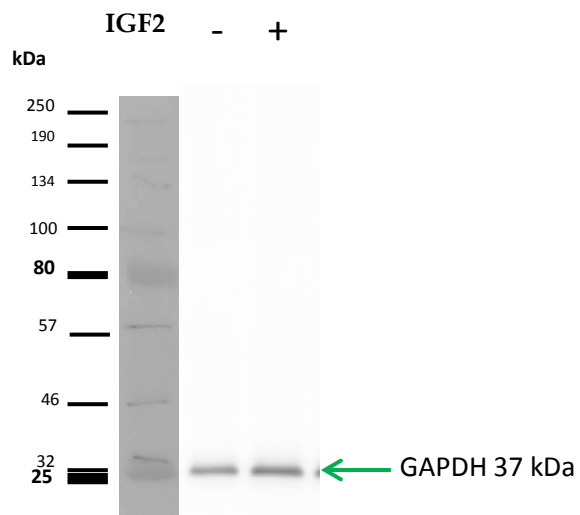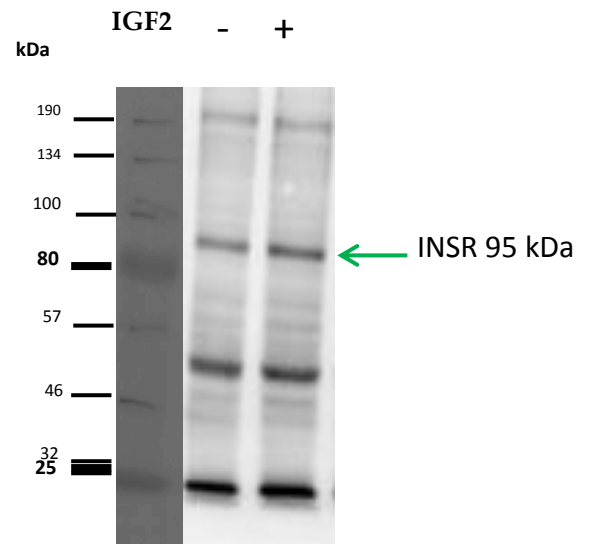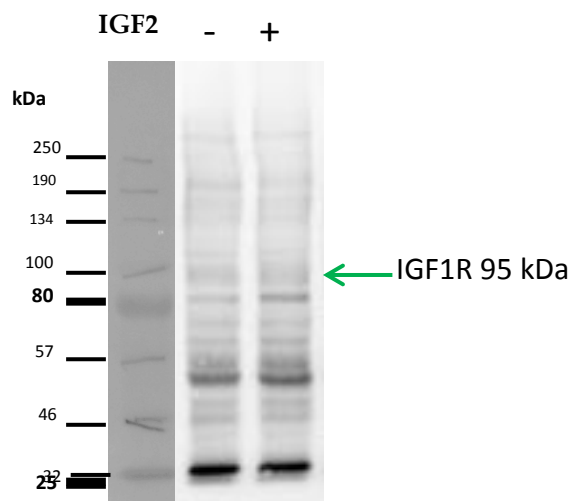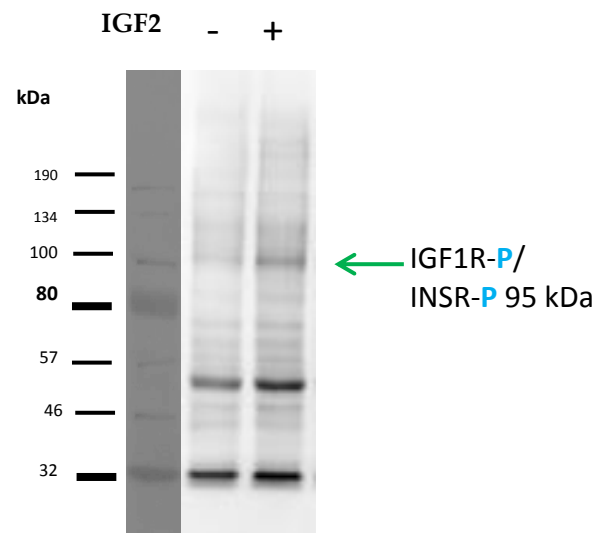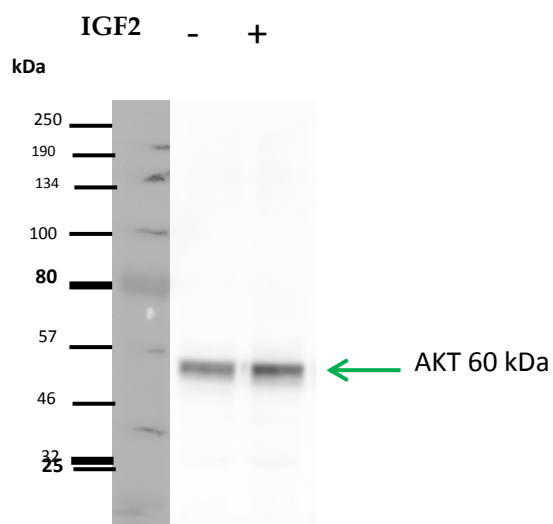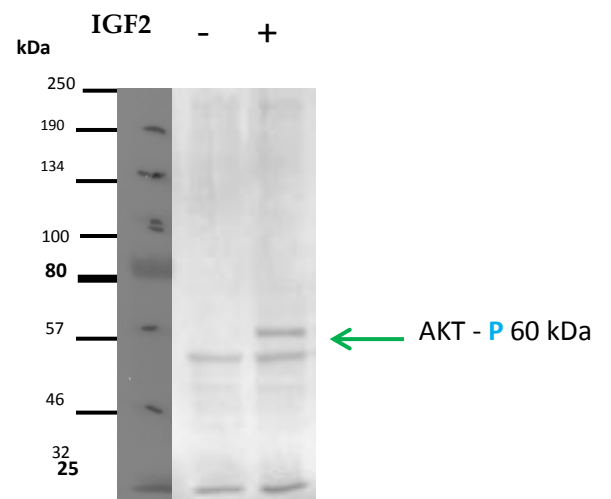

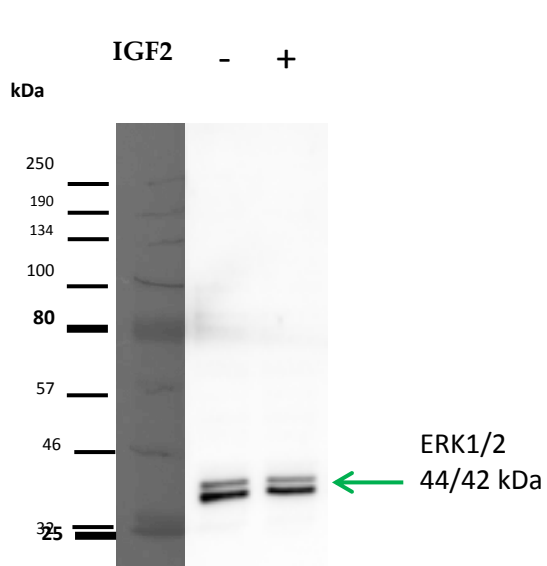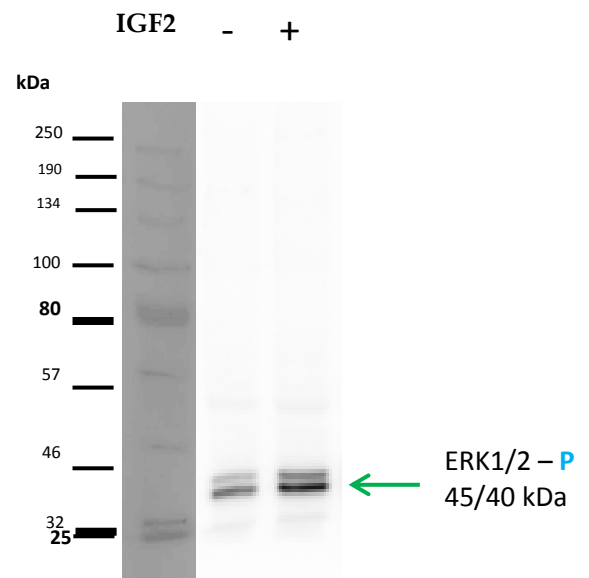

ERK 1/ERK 2 (44/42  
kDa) was stained  
before

P- ERK 1/ERK 2 (45/40  
kDa) was stained  
before

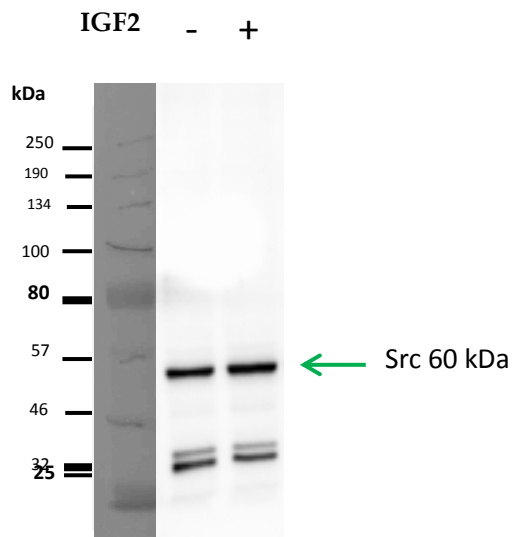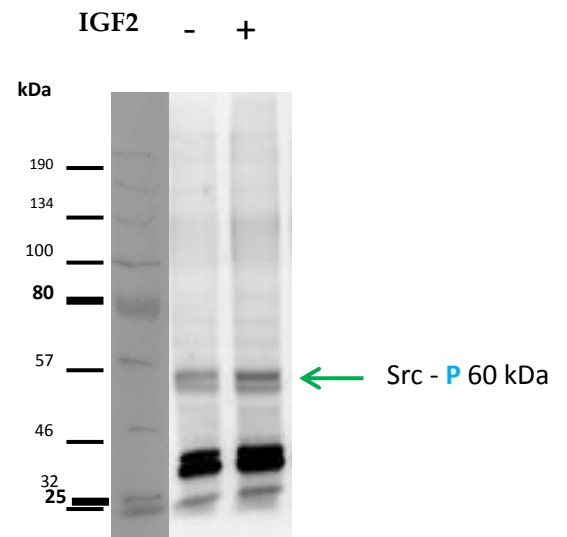

Supplement: Supplementary file 1 [file cancers-12-00793-s001.zip › cancers-744773 supplementary final/cancers-744773 non published materials.pdf]
